# Economic Analysis of Integrating Digital Cognitive Assessments and Blood‐Based Biomarker Testing in Alzheimer's Screening Workflow

**DOI:** 10.1002/alz70860_098541

**Published:** 2025-12-23

**Authors:** Yi Sun

**Affiliations:** ^1^ University of Reading, Reading, Reading, United Kingdom

## Abstract

**Background:**

The rising prevalence of dementia and the growing availability of disease‐modifying therapies (DMTs) emphasize the need for efficient and scalable diagnostic pathways. Current health system constraints in capacity and accessibility pose challenges in identifying patients eligible for DMTs. An at‐home integrated digital cognitive assessment including speech, EEG test via wearable devices and Mini Mental State Examination (MMSE) equivalents can support primary care in their role as a ‘gatekeeper’, ensuring more effective referrals to memory clinics. Also, blood‐based biomarker (BBBM) tests offer a promising solution for improving patient stratification at the primary care level. To explore these innovations, we will conduct a health system simulation study to evaluate the impact of combining the integrated digital assessments and Blood‐based biomarker test on diagnostic costs and wait times within the healthcare system.

**Method:**

We will employ a Markov decision‐analytic model to simulate three stratification scenarios for patients at the primary care level: (1) use cognitive and physical activity assessment only; (2) implement the integrated digital cognitive assessments at home, followed by cognitive and physical activity assessment if needed; (3) use the at‐home integrated digital cognitive assessment, followed by cognitive and physical activity assessment and blood‐based biomarker test if required. The analysis will adopt the perspective of the healthcare system and compare these scenarios based on resource utilization, costs, and diagnostic outcomes.

**Results:**

We planned to report and compare the average annual cost, average waiting time for completion of the diagnostic process, and rates of correct case identification across the three scenarios.

**Conclusions:**

The combination of the integrated digital cognitive assessments and blood‐based biomarker test may alleviate pressure on general practitioners and improve patient access to timely care. In future analysis, we will conduct direct empirical validations using cohort data.

